# Automated analysis of whole slide digital skin biopsy images

**DOI:** 10.3389/frai.2022.1005086

**Published:** 2022-09-20

**Authors:** Shima Nofallah, Wenjun Wu, Kechun Liu, Fatemeh Ghezloo, Joann G. Elmore, Linda G. Shapiro

**Affiliations:** ^1^Department of Electrical and Computer Engineering, University of Washington, Seattle, WA, United States; ^2^Department of Biomedical Informatics and Medical Education, University of Washington, Seattle, WA, United States; ^3^Paul G. Allen School of Computer Science and Engineering, University of Washington, Seattle, WA, United States; ^4^David Geffen School of Medicine, University of California Los Angeles (UCLA), Los Angeles, CA, United States

**Keywords:** whole slide image, melanocyte, melanoma, mitosis, semantic segmentation, deep learning, viewing patterns, diagnosis

## Abstract

A rapidly increasing rate of melanoma diagnosis has been noted over the past three decades, and nearly 1 in 4 skin biopsies are diagnosed as melanocytic lesions. The gold standard for diagnosis of melanoma is the histopathological examination by a pathologist to analyze biopsy material at both the cellular and structural levels. A pathologist's diagnosis is often subjective and prone to variability, while deep learning image analysis methods may improve and complement current diagnostic and prognostic capabilities. Mitoses are important entities when reviewing skin biopsy cases as their presence carries prognostic information; thus, their precise detection is an important factor for clinical care. In addition, semantic segmentation of clinically important structures in skin biopsies might help the diagnosis pipeline with an accurate classification. We aim to provide prognostic and diagnostic information on skin biopsy images, including the detection of cellular level entities, segmentation of clinically important tissue structures, and other important factors toward the accurate diagnosis of skin biopsy images. This paper is an overview of our work on analysis of digital whole slide skin biopsy images, including mitotic figure (mitosis) detection, semantic segmentation, diagnosis, and analysis of pathologists' viewing patterns, and with new work on melanocyte detection. Deep learning has been applied to our methods for all the detection, segmentation, and diagnosis work. In our studies, deep learning is proven superior to prior approaches to skin biopsy analysis. Our work on analysis of pathologists' viewing patterns is the only such work in the skin biopsy literature. Our work covers the whole spectrum from low-level entities through diagnosis and understanding what pathologists do in performing their diagnoses.

## 1. Introduction

Skin cancer is the most common type of cancer. The main types of skin cancer are squamous cell carcinoma, basal cell carcinoma, and melanoma. Melanoma is much less common than the other types, but much more likely to invade nearby tissue and spread to other parts of the body. Most deaths from skin cancer are caused by melanoma. Melanoma usually begins in melanocytes, which are specialized cells that make melanin (the pigment that gives skin its color). The current gold standard for melanoma diagnosis is the pathologists' assessment after microscopic viewing examination of skin biopsies using hematoxylin and eosin (H&E) stained tissue sections; however, the histologic interpretation of melanocytic lesions is challenging with pathologists' diagnosis noted to be neither accurate nor reproducible (Elmore et al., 2017). Whole slide digital imaging of pathology specimens can be used to create digitized slides, which in turn can be included in biorepositories or used in telepathology to enable diagnosis at a distance. By investigating the potential to improve diagnoses using digitized slides and associated image characteristics, we show that artificial intelligence can provide clinical support for pathologists. This paper provides an overview of multiple efforts by our research group to this end. Our work differs from prior work on melanoma biopsy analysis in its use of deep learning as the major tool. Our contributions include:

- A new mitosis detection method that compares two deep learning architectures and has been tested on both skin biopsies and breast cancer biopsies,
- A new melanocyte detection method that uses a Generative Adversarial Network (GAN) to generate synthetic SOX10-stained images and is novel in its use of the features of the GAN decoder for the melanocyte detection,
- A new deep learning-based semantic segmentation system that segments a whole slide skin biopsy image into important components, such as the epidermis, the dermis, and the nests within them, for use in diagnosis,
- A scale-aware transformer system that can diagnose whole slide skin biopsy images using multiple scales and is the first such deep learning system to do so,
- A thorough analysis of the viewing patterns of a group of practicing pathologists who diagnosed digital whole slide skin biopsy images using a web-based viewing platform in order to understand what variables affected their diagnoses.

Our work is novel in its use of advanced deep learning architectures to tackle a set of problems in the skin biopsy analysis domain that together can lead to a full diagnostic aid system. Limitations include the use of only a single curated data set, since there were no publicly available data sets, and that we have not yet integrated all the above work into an end-to-end system. Such work is still underway.

## 2. Related work

While there has been a great deal of work in other areas of pathology, such as breast cancer pathology, there is little work in computer analysis of skin cancer biopsies. This is due to the lack of public data sets with ground truth available, so researchers have to collect their own data. Our work was preceded by some groundbreaking work at the University of Alberta in Professor Mrinal Mandal's group in which all topics we address, from finding low-level entities to diagnosing the images, was pursued. Lu worked on melanocyte detection (Lu et al., 2013a) and mitotic cell detection (Lu and Mandal, 2014b) as well as segmentation and analysis of the epidermis (Lu and Mandal, 2014a). He also developed the first diagnosis system (Lu and Mandal, 2015). Hong Xu created a full system that segmented the epidermis and dermis (Xu and Mandal, 2015), analyzed the features of both of them from a computer-vision-feature point of view, and used these features to perform diagnosis (Xu et al., 2018).

Our work follows these studies but has substantial differences. The Alberta work was feature-based, while our work is deep-learning based. Their hand-crafted features for finding melanocytes (the halo approach) (Lu et al., 2013a) did not transfer well on our data set. To capture the complexity of skin biopsy images, we have divided our work into three sections: finding low-level entities, semantic segmentation of the images, and diagnosis. We also study the association of pathologists' viewing behavior with diagnostic accuracy.

## 3. Low-level entity detection

### 3.1. Methods

There are several low-level entities to be found in skin biopsy images that are clinically useful to pathologists when making their diagnoses, and we have developed methods for finding two of them: (1) melanocytes, which can be found alone or in nests, and (2) mitotic figures, which are cells in a biopsy that are actively dividing into two cells, indicating that the diagnosis could be more severe.

#### 3.1.1. Melanocyte detection and results

**Figure 1:**
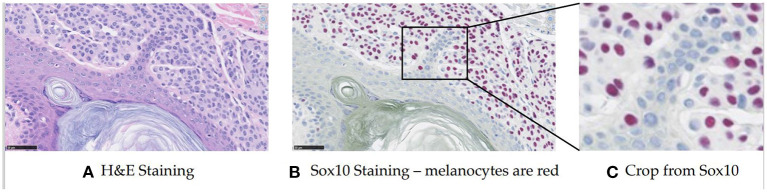
Sample H&E stained image and SOX10 stained image. The SOX10 stain highlights the melanocytes in red-brown (This figure appears in a manuscript submitted to WACV 2023). **(A)** H&E staining. **(B)** Sox10 staining—melanocytes are red. **(C)** Crop from Sox10.

Melanocytes are cells that produce and contain the pigment called melanin, which protects against ultraviolet radiation. They normally reside in the basal layer of the epidermis, but in abnormal biopsies, they can be found in multiple different locations and the distribution disorder is important for diagnosis. In prior work (Lu et al., 2013a,b), a feature-based method was proposed based on the assumption that melanocytes were cells surrounded by a halo appearance. However, this turned out to not apply to some melanocytes in our preliminary work. Thus we took a different approach, using deep learning for melanocyte detection. To enable supervised learning and have a fair evaluation, we began to look at specific immunohistochemical (IHC) stainings that could highlight the melanocytes (the SOX10 stain, for example, turns the melanocytes red-brown) (see Figure 1). Since the SOX10 stain is not routinely used in both clinical practice and computer vision study, we wanted to keep it as an auxiliary reference to facilitate melanocyte detection and only take H&E images as input. With this in mind, we first trained the ESPNetV2 (Mehta et al., 2019) to classify pre-segmented nuclei patches. However, this deep learning classifier was not satisfactory due to the visual similarity of melanocytes and other cells. Inspired by the application of Generated Adversarial Networks (GANs) in virtual staining (Xu et al., 2019; Liu et al., 2021), we assume the mapping between H&E and SOX10 can be learned and can facilitate melanocyte detection. Thus we propose VSGD-Net, a virtual staining guided detection network, which learns melanocyte identification through virtual staining from H&E to SOX10.

**Figure 2:**
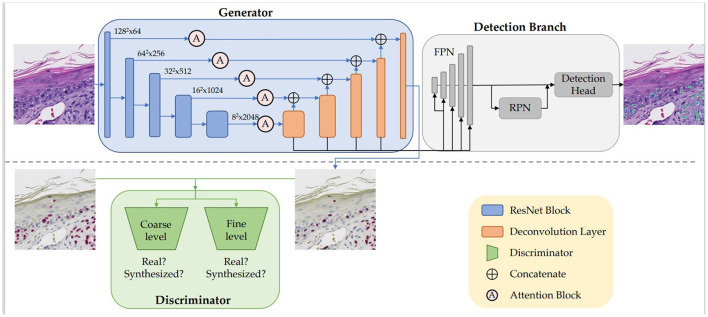
Our VSGD-Net framework: H&E images are virtually stained to SOX10. The jointly trained detection branch utilizes the intermediate features in the generator to detect melanocytes and provides feedback to the generator to enhance synthesis quality. The inference phase only uses the upper part of the architecture (This figure appears in a manuscript submitted to WACV 2023).

Figure 2 illustrates the architecture, which is comprised of the generator *G*, the discriminator *D*, and the detection branch *Det*. We built the generator *G* based on an adapted UNet (Ronneberger et al., 2015) structure with ResNet-50 (He et al., 2016) being the backbone. The encoder-decoder structure learns the high-dimensional feature representation of input H&E images, and translates them into target SOX10 stained images. We incorporated attention blocks (Woo et al., 2018) in the skip connections between the encoder and the decoder. While the generator learns the virtual staining process, the discriminator attempts to differentiate real and synthesized SOX10 images. Inspired by Pix2PixHD (Wang et al., 2018), we adopted a multi-scale architecture that has 2 identical CNN networks as discriminators: the two discriminators work at coarse and fine levels separately, where the input to the coarse-level discriminator is down sampled by a factor of 2 from the input to the fine-level discriminator. Optimized by the minimax loss (Goodfellow et al., 2014), the intermediate features in *G* contain the hidden correlation between H&E and SOX10, thus can be exploited to detect melanocytes. Similar to Mask R-CNN (He et al., 2017), our detection branch *Det* contains a feature pyramid network (FPN), a region proposal network (RPN), and the downstream heads. As the SOX10 stain can highlight melanocytes, we placed *Det* after the decoder of *G*, which is closer to the SOX10 stain. *G*, *D*, and *Det* are trained jointly to simultaneously learn from the image synthesis and the detection tasks.

We compared our VSGD-Net with a nuclei classification method performing melanocyte detection using ESPNetv2 (Mehta et al., 2019), and a group of nuclei detection methods performing melanocyte detection including Radial Line Scanning (RLS) (Lu et al., 2013b), Mask R-CNN (He et al., 2017), U-Net (Ronneberger et al., 2015), HoverNet (Graham et al., 2019), and the state-of-the-art CHR-Net (Gao et al., 2021). Precision, Recall, F1-score, and Jaccard index were reported on the test set. As Table 1 shows, VSGD-Net achieved the best F1-score and Jaccard index by balancing the performance between precision and recall.

**Table 1:** Comparison with nuclei detection methods for melanocyte detection (This table appears in a manuscript submitted to WACV 2023).

**Method**	**Precision**	**Recall**	***F*_1_**	**Jaccard**
Nuclei classification	0.693	0.506	0.585	0.413
RLS (Lu et al., 2013b)	0.443	0.570	0.499	0.332
Mask R-CNN (He et al., 2017)	**0.735**	0.514	0.605	0.434
U-Net (Ronneberger et al., 2015)	0.630	0.639	0.635	0.465
HoverNet(Graham et al., 2019)	0.729	0.499	0.592	0.421
CHR-Net (Gao et al., 2021)	0.607	0.688	0.645	0.476
**Ours VSGD-Net**	0.660	**0.710**	**0.684**	**0.520**

The bold values are the best scores in each column.

#### 3.1.2. Mitotic figure detection and results

A mitosis (or mitotic figure) is an important entity in the review of skin biopsy cases as its presence may aid in the diagnosis of a melanoma in addition to being associated with poorer prognosis. A high mitotic rate in a primary invasive melanoma is associated with a lower survival probability. Among the independent predictors of melanoma-specific survival, mitotic rate is the strongest prognostic factor after tumor thickness (Thompson et al., 2011). Thus, the accurate detection of mitotic activity plays an important role in making cancer diagnoses for the pathologist, and because mitoses are small objects with various shapes that can resemble normal nuclei, mitosis detection remains a challenging task for humans.

Various approaches have been applied to detect mitotic figures. For example, Irshad et al. used morphological features to identify cellular entities in a breast biopsy dataset (Irshad et al., 2013). Cireşan et al. (2013) used a CNN-based method for mitosis detection and won the International Conference on Pattern Recognition 2012 (ICPR 2012) mitosis detection challenge by a significant margin. Since then, much of the research on mitosis detection in breast cancer biopsy images has used CNNs, and CNN-based methods have been proposed for mitosis detection in different tissues, including breast, stem cells, and skin. To the best of our knowledge, there are no publicly available skin biopsy datasets with mitosis annotations from experienced dermatopathologists. To conduct our research on mitotic figures in skin biopsy images, we created a new dataset with mitosis-level markings from an expert pathologist. We studied and compared the performance of two different state-of-the-art CNNs, one that is small and was designed for use in low-capacity devices and one that is much bigger, in terms of accuracy, sensitivity, specificity, precision, recall, and F-score. Our research, in general, gives a methodology and architecture for mitosis finding in both melanoma and breast cancer whole slide images, and that is likely to be useful for finding mitoses in any whole slide biopsy images (Nofallah et al., 2021) .

An expert pathologist (S. Knezevich) chose six skin biopsy cases of ≥ T1b invasive melanoma, from our dataset and cropped 34 areas in the whole slide images (WSIs) of these cases. The size of the areas and the number of areas per each case were not fixed but were based on the pathologist's judgment with the aim of marking as many mitoses as possible. A total of 628 mitoses in the cropped image areas were marked by the same pathologist using the Sedeen Viewer. These marked mitoses provide “class mitosis” samples for training and validation of our binary classifiers.

**Figure 3:**
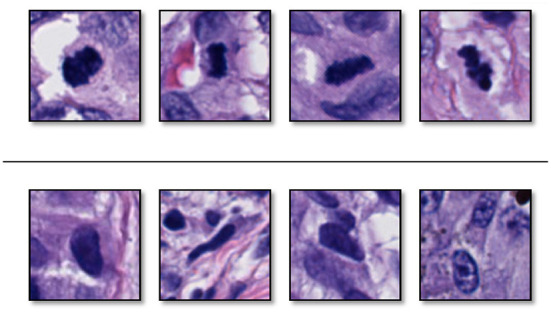
Examples of **(top)** sampled mitoses, and **(bottom)** sampled nuclei that are not mitoses. These two entities have similarity in color, surrounding, and texture (Reprinted by permission from Elsevier, Computerized Medical Imaging and Graphics, “Machine learning techniques for mitoses classification,” S. Nofallah, S. Mehta, E. Mercan, S. Knezevich, C. J. May, D. Weaver, D. Witten, J. G. Elmore, L. G. Shapiro, Copyright Elsevier 2021.).

Figure 3 shows some examples of mitoses and normal nuclei. This figure shows the similarity between these entities in terms of texture, color, and shape. We used a 101 × 101 patch approximately centered on the target entity's center. To help our classifier learn rotation, scale, and translation-invariant representations, we augmented our training set with standard augmentation methods such as rotation (45, 90, 135 or 225 degrees) and mirroring (horizontal and vertical). The original images were padded on the borders.

Our classification network uses a standard pipeline (Krizhevsky et al., 2012; He et al., 2016) that stacks encoding and down-sampling units to learn latent representations. In our experiments, we used two state-of-the-art encoding units: (1) Efficient Spatial Pyramid of Dilated Convolutions (ESPNet) (Mehta et al., 2018b) and (2) Densely Connected Convolutional Networks (DenseNet) (Huang et al., 2017). The same dataset split (training 80%, validation 20% with 3:1 ratio for normal vs. mitosis) was used for both ESPNet and DenseNet training and validation.

Table 2 summarizes the results of our classifiers using two different encoding units: 1) ESPNet and 2) DenseNet. Both networks achieved high accuracy on classifying mitoses with a sensitivity of 0.976 and 0.968, and specificity of 0.987 and 0.995, respectively. Though DenseNet outperformed ESPNet, this outperformance was not statistically significant (*p*-value is 0.5716), and the training time of ESPNet is about a third that of DenseNet.

**Table 2:** Quantitative results of ESPNet and DenseNet on validation set for mitosis detection (This table was adapted from Nofallah et al., 2021).

**Metrics**	**ESPNet**	**DenseNet**
Accuracy	0.984	0.988
Precision	0.961	0.984
Recall	0.976	0.968
Sensitivity	0.976	0.968
Specificity	0.987	0.995
FP, FN	5, 3	2, 4
TP, TN	122, 370	121 , 373
Training time	35 m and 6 s	106 m and 32 s

### 3.2. Discussion

In a pathologist's decision-making process, the distribution disorder of melanocytes on whole slide images is a key factor for melanoma diagnosis. As shown by Table 1, our VSGD-Net successfully detects melanocytes from only the routine H&E staining. Although the state-of-the-art results of the melanocyte detection in both *F*_1_-score and Jaccard index are below 0.7, they are sufficient to reveal an estimated melanocyte distribution, given the over 70% recall score. Our melanocyte detector is useful to show pathologists without their having to obtain additional immunohistochemical stained images of the biopsy material with the corresponding time and expense.

The results of mitotic figure classification, given a good cell finder, are very good. While the change from the 7th (Edge and Compton, 2010) to the 8th (Amin et al., 2017) edition of the American Joint Committee on Cancer (AJCC) cancer staging system for melanoma no longer includes information on the presence of mitotic figures as criteria for defining melanoma stages, the presence of mitotic cells remains an important prognostic feature in clinical practice.

Currently, our low-level feature detection systems are stand-alone, but they can also be integrated into the higher-level systems we are designing.

## 4. Semantic segmentation

Semantic segmentation refers to the classification of the pixels of a WSI into categories, in our case the tissue classes of each pixel. Accurate semantic segmentation has the potential to improve the performance of automated diagnosis systems or help pathologists reduce classification uncertainties. However, a major problem of these methods is the lack of labeled ground-truth data because pixel-level labeling of gigapixel WSIs is extremely time-consuming and must be done by expert pathologists.

### 4.1. Method

To reduce the burden of extensive label acquisition, in the work of Nofallah et al. (2022b), we introduce a simple two-step approach for semantic segmentation of skin biopsy WSIs using coarse and sparse labels, which can significantly reduce the labeling cost. Skin biopsy images have entities of variable size. Entities like the dermis and epidermis are large and easy to segment (Xu and Mandal, 2015), while entities like dermal and epidermal nests are small and more difficult to segment. When we have sparser annotations on smaller entities, training a segmentation model that has high accuracy on all the tissue structures might be harder to achieve. Hence, if the segmentation model is trained in a single-stage with all the labels at once, the model will perform better on larger entities and not as well on the smaller ones.

**Figure 4:**
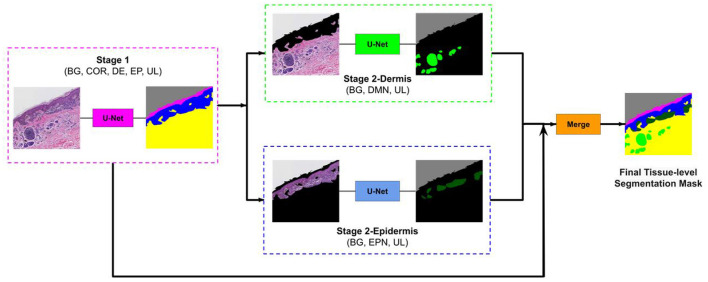
Overview of our approach. The image first goes to stage 1 and the segmentation mask of entities (COR, Stratum Corneum; EP, Epidermis; DE, Dermis; BG, Background; UL, Unlabeled) in stage 1 is generated. Then this mask is used to remove the epidermis from stage 2-Dermis input and remove the dermis from stage 2-Epidermis input. The modified images are fed to their corresponding trained model. Stage 2-Dermis generates the segmentation masks of entities present in the dermis (DMN, Dermal nests), and stage 2-Epidermis generates the entities in the epidermis (EPN, Epidermal nests). In the end, stage 2-Dermis and stage 2-Epidermis segmentation masks are overlaid on the stage 1 mask and the final tissue-level segmentation mask is generated (Reprinted by permission from Springer, Journal of Digital Imaging, “Segmenting Skin Biopsy Images with Coarse and Sparse Annotations using U-Net,” S. Nofallah, M. Mokhtari, W. Wu, S. Mehta, S. Knezevich, C. J. May, O. H. Chang, A. C. Lee, J. G. Elmore, L. G. Shapiro, Copyright 2022.).

To overcome this problem, we developed a two-stage segmentation pipeline: First, a segmentation U-Net model is trained with labels of large entities in the histopathology image (Background, Stratum Corneum, Epidermis, Dermis). Then, in the second stage, there are two sub-stages: (1) Stage 2-Dermis is trained on the dermis portion of the images and uses the ground truth for the smaller entities that are present in Dermis (i.e., Dermal nests). (2) Stage 2-Epidermis is trained on the epidermis portion of the images and uses the ground truth for the smaller entities that are present in Epidermis (i.e., Epidermal nests). Figure 4 illustrates the structure of our segmentation system.

### 4.2. Results

**Figure 5:**
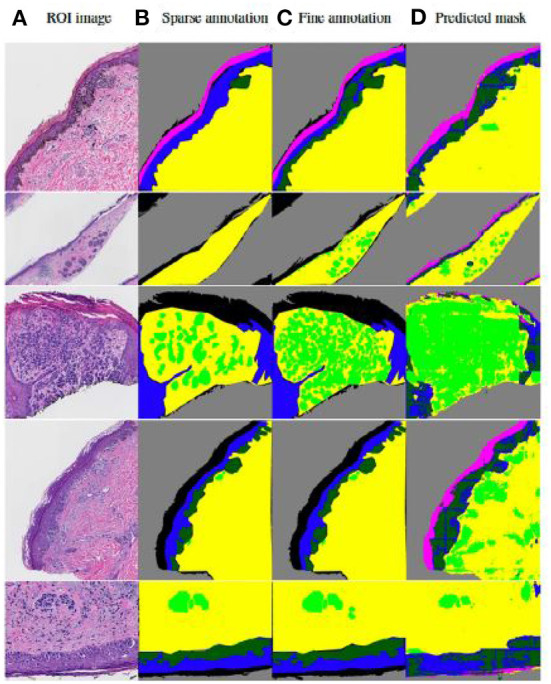
Visualization of results by our published two-step segmentation method (Nofallah et al., 2022b). **(A)** Examples of original image; **(B)** Sparse expert annotation; **(C)** Fine-detailed epidermal and dermal nest annotation by expert for evaluation; **(D)** Segmentation results by our published work. The annotation and segmentation images contain the *dermis* (*DM*-yellow), *epidermis* (*EP*-blue), *stratum corneum* (*COR*-pink), *background* (*BG*-gray), *dermal nests* (*DMN*-light green), and *epidermal nests* (*EPN*-dark green).

Tissue structures used in this study are: *background* (*BG*), *epidermis* (*EP*), *dermis* (*DM*), *stratum corneum* (*COR*), *epidermal melanocytic nest* (EPN), and *dermal melanocytic nest* (*DMN*). Figure 5 demonstrates some examples of input images, the corresponding sparse annotations, the corresponding fine-detailed annotations on *DMN* and *EPN* by clinical experts in dermatopathology, and the segmentation results of our approach. Table 3 shows quantitative results on regions of interest (ROIs), for which we had pathologists mark the structures in the test set. Results show that our method can generate high-quality segmentations for *DM* and *EP*, while over-labeling *DMN* and *EPN*, an expected problem of a small dataset with noisy ground-truth annotations. Despite the imperfect results, these clinically-relevant tissue segmentations can still be informative to improve diagnostic performance, which is shown in Nofallah et al. (2022a).

**Table 3:** Evaluation of the segmentation model on ROI testing set (This table appeared in Nofallah et al., 2022b).

**Segmentation stage**	**Dice score**	**IoU**
Stage 1 (all tissues)	0.942	0.906
Stage 2-Dermis (DMN)	0.558	0.638
Stage 2-Epidermis (EPN)	0.332	0.558

### 4.3. Discussion

While segmentation is a significant element in the diagnosis pipeline, training a segmentation model generally requires a large, high-quality annotated ground-truth set. However, one of the biggest challenges in dealing with most medical datasets is acquiring sufficiently-sized and carefully-annotated datasets, since the standard ground-truth on these datasets is expert-level annotation, which is a challenging, time-consuming, and expensive task. In our segmentation work, we proposed a two-stage pipeline for the segmentation of important tissue structures in skin biopsy images using coarse and sparse annotations on small regions of WSIs. Our system was able to generate segmentation masks for both epidermis/dermis and nests with high-quality performance, indicating that having sparse annotation on important tissues has the potential for producing a useful segmentation model.

## 5. Diagnosis

For a reliable diagnostic system, it is important to obtain representations that reflect both the content and context of the input biopsy image. HATNet, a system we developed originally for breast biopsy analysis, achieved this using a top-down and bottom-up approach (Mehta et al., 2022). Pathologists describe using a different viewing behavior before making their diagnosis of breast tissue compared with their assessment of skin biopsy images. Pathologists often examine features of various tissues, such as skin biopsies, by changing the magnification of a microscope back and forth. Our methodology for skin biopsies, called ScAtNet, is motivated by pathologists' viewing behavior.

### 5.1. Method

**Figure 6:**
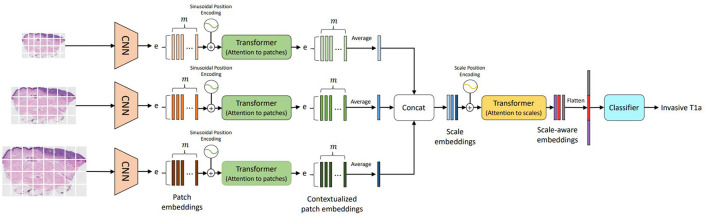
Overview of ScAtNet for classifying skin biopsy images (Wu et al., 2021). To learn representations from these large WSIs at multiple input scales in an end-to-end fashion, ScAtNet factorizes the classification pipeline into three steps. The first step involves learning local patch-wise embeddings using an off-the-shelf CNN for each input scale independently. In the second step, ScAtNet learns inter-patch representations using transformers and produces contextualized patch embeddings for each input scale. In the last step, ScAtNet learns inter-scale representations from concatenated multi-scale contextualized patch embeddings using another transformer network and produces scale-aware embeddings, which are then classified linearly into diagnostic categories (This figure was published in Wu, W., Mehta, S., Nofallah, S., Knezevich, S., May, C. J., Chang, O. H., Elmore, J. G., Shapiro, L. G. (2021), “Scale-aware transformers for diagnosing melanocytic lesions,” IEEE Access 9, 163526–163541, under a Creative Commons License.).

Following (1) the success of transformers in vision (Vaswani et al., 2017), (2) the methods for learning representations from different input scales (Chen et al., 2016; Lin et al., 2017; Mehta et al., 2018a), and (3) the importance of input scales for diagnosis in clinical settings (Brunyé et al., 2017; Mercan et al., 2018), we introduced a self-attention-based deep neural network called the Scale-Aware Transformer Network (ScAtNet) that adapts to the information from different input scales using self-attention and predicts the classification label (Wu et al., 2021). Figure 6 shows an overview of ScAtNet, which has three main steps: (1) learn local patch-wise embeddings using a CNN for each input scale, (2) learn contextualized patch-embeddings for each input scale using transformers, and (3) learn scale-aware embeddings across multiple input scales using transformers.

The input WSI image is divided into *m* non-overlapping patches. Patch-wise feature representations, referred to as patch embeddings, are obtained using an off-the-shelf CNN. The patch embeddings are produced *independently* for each patch. In other words, these embeddings do not encode inter-patch relationships. They are fed to a transformer to learn inter-patch relationships. Similar to vision transformers (Dosovitskiy et al., 2021), patch-wise sinusoidal positional embeddings are added to the original embeddings to encode the position of input patches. The resultant embeddings are then fed to a transformer to produce contextualized patch embeddings. These contextualized embeddings are then averaged along the *m*-dimension to produce an *e*-dimensional embedding vector, which encodes the local (from CNN) and global (from Transformer) information in an image **X**^*sc*^.

A contextualized patch embedding encodes the information in an image **X**^*sc*^ at scale *sc*. Let us assume that we have S scales. For each scale *sc*∈[0, ..., S], we produce an embedding vector and concatenate them all to produce a scale-level embedding vector. These embeddings do not encode information about the relationships between the different scales. To learn scale-aware representations while retaining positional information about each scale, scale-level learnable positional embeddings are added. The resultant embeddings are then fed to another transformer to produce contextualized scale embeddings. For predicting the diagnostic class, ScATNet first flattens the scale-aware embeddings to produce a (*sc*·*e*)-dimensional vector and then classifies it using a linear classifier into *C* diagnostic categories.

### 5.2. Results

Table 4 compares the overall performance of ScAtNet across different metrics on single- and multi-scale inputs. Two scales improved the performance over only one scale. Compared to two scales, the overall performance with three scales remains the same. However, with three scales, the performance across all diagnostic classes was much more evenly distributed, which is not seen in all other combinations. Our experiments also show that our method outperforms five other state-of-the-art whole slide image classification methods by a significant margin. Our method also achieves comparable performance to 187 practicing U.S. pathologists who interpreted the same cases in an independent study.

**Table 4:** Overall performance of ScAtNet (This table was published in Wu, W., Mehta, S., Nofallah, S., Knezevich, S., May, C. J., Chang, O. H., Elmore, J. G., Shapiro, L. G. (2021), “Scale-aware transformers for diagnosing melanocytic lesions,” IEEE Access 9, 163526–163541, under a Creative Commons License.).

**Input scales**				**Accuracy**	**F1**	**Sensitivity**	**Specificity**	**AUC**
**7.5 × **	**10 × **	**12.5 × **						
✓				0.55	0.55	0.55	0.85	0.75
	✓			0.60	0.60	0.60	0.87	0.77
		✓		0.61	0.61	0.61	0.87	0.78
✓	✓			0.64	0.64	0.64	0.88	0.79
✓		✓		0.63	0.63	0.63	0.88	0.80
	✓	✓		0.63	0.63	0.63	0.88	0.79
✓	✓	✓		0.63	0.63	0.63	0.88	0.79

### 5.3. Discussion

Unlike prior studies, this work classifies the full spectrum of melanocytic skin biopsy lesions ranging from mildly atypical nevi and more advanced atypical pre-cursor lesions, to melanoma *in situ* to invasive melanoma. An independent test set allows us to demonstrate the generalization ability of ScATNet. A key strength of our work is that we were able to compare the diagnostic classification of ScATNet with the performance of actively practicing U.S. pathologists who interpreted the same cases (test set) in an independent study.

Although the proposed method has shown great potential for automated melanocytic lesion classification, limitations are recognized. Our study is only relevant to melanocytic lesions, while only about one in four skin biopsies have melanocytic cells (Lott et al., 2018). Moreover, despite having an independent test set, ScATNet was evaluated on only 115 WSIs. In order to demonstrate its application in clinical settings, it should be tested on a larger test set. Also, in this paper, we only studied skin biopsies. However, we believe that ScATNet is generic and can be extended to other types of biopsy images, such as breast and lung.

## 6. Association of pathologists' viewing behaviors with diagnostic accuracy

Making a diagnosis from a pathology slide is a difficult process that requires years of training for pathologists. A pathologist typically uses a microscope to examine a skin sample on a glass slide in an effort to identify important areas and visual characteristics. These characteristics can be subtle and difficult to understand, but they have important implications for diagnosis and treatment at the regional and cellular levels. When faced with massive amounts of information on huge slides, even experienced pathologists can make mistakes. Studies have shown that even when pathologists see the same characteristics on a biopsy sample slide, their diagnoses can differ (Zhang et al., 2019). Since digital imaging is becoming more prevalent in diagnostic pathology, it is crucial to study the pathologists' viewing behaviors as they diagnose a digital image. The outcomes of these research efforts can be helpful in a number of areas, including enhancing the education and training of pathologists, identifying the causes of diagnostic errors to improve pathologists' performance, and assisting in the creation of computer-aided diagnostic tools.

### 6.1. Methods

To gain a better understanding of pathologists' viewing behaviors, we introduce various ways of quantifying such behaviors while the pathologist is viewing and diagnosing digital whole slide images (Ghezloo et al., 2022). Then we investigate how these viewing behaviors are associated with their diagnostic accuracy and pathologist characteristics and demographics. In our study, 32 pathologists used a web-based viewer to examine and diagnose one of five sets of 36 digital melanocytic skin cases (180 total cases) that were assigned to them. These viewing sessions were recorded, producing a total of 1073 interpretations. The web-based viewing platform automatically recorded a series of viewports in the order in which they were viewed. A viewport is a rectangular area of the image that can be seen at any time during interpretation on the pathologist's computer screen. The tracking data for each interpretation included the location of the viewports, the zoom level used to see the viewports, and the timestamps. Additionally, we have a consensus reference diagnosis and a ROI (region of interest) for each digital case that highlights key aspects of the diagnosis as determined by our reference panel of expert pathologists.

Using the viewport tracking data, we defined variables to measure pathologists' viewing behaviors regarding their interactions with the digital slides, including their zooming and panning habits, total interpretation time, and attention to the reference panel's chosen consensus ROI. A list of these variables and their definitions can be found in Table 5. Total interpretation time measures the amount of time a pathologist spends viewing a digital slide. To evaluate pathologists' zooming behavior, we measured the average, maximum and variance of zoom levels used during an interpretation. ROI time percentage measures the percentage of total interpretation time spent viewing areas that intersect with our panel of experts' selected ROIs. For a better understanding of zooming behavior, we defined magnification percentage, which measures the percentage of viewports where a pathologist zooms in consecutively, and scanning percentage, where a pathologist utilizes a fixed zoom level to scan the slide.

**Table 5:** Pathologists' viewing behaviors and their association with diagnostic accuracy (This table was published in Journal of Pathology Informatics, Vol 13, F. Ghezloo, P. Wang, K. F. Kerr, T. T. Brunye, T. Drew, O. H. Chang, L. M. Reisch, L. G. Shapiro, J. G. Elmore, “An analysis of pathologists' viewing processes as they diagnose whole slide digital images,” 1–6, Copyright Elsevier (2022).).

**Viewing behavior** **(Predictor variable)**	**Adjusted OR (95% CI)**	***P*-value**
Total interpretation time	1.33 (1.09, 1.62)	0.005
Average zoom	1.26 (1.03, 1.54)	0.023
Maximum zoom	1.24 (1.03, 1.50)	0.026
Zoom variance	1.37 (1.11, 1.68)	0.003
Magnification percentage	0.76 (0.63, 0.92)	0.006
ROI time percentage	1.35 (1.07, 1.69)	0.011
Scanning percentage	1.21 (1.00, 1.47)	0.054

We applied a generalized linear mixed model with logit link that converts values to a 0-1 scale to examine the associations between pathologists' viewing behavior and diagnostic accuracy on each case. The binary agreement between a pathologist's diagnosis and the expert-defined consensus reference diagnosis is how we define diagnostic accuracy. We utilized one of the viewing behaviors defined above as the explanatory variable of interest and diagnostic accuracy as the outcome for each univariate model. All models also included the pathologists' years of experience with melanocytic skin lesions and their board certification and/or fellowship training to control for pathologist experience or expertise.

### 6.2. Results

For each of the defined viewing behaviors, seven different models were created. Table 5 displays the Odds Ratio (OR) and *P*-value for each model. With a viewing behavior as the predictor variable and diagnostic accuracy as the outcome, each row represents one model. Except for scanning percentage, which was marginally significant ( 0.05 < *P*< 0.1), all viewing behaviors exhibit a statistically significant association with diagnostic accuracy (*P* < 0.05). Each viewing behavior, with the exception of magnification percentage, was positively correlated with accuracy (*adjusted* *OR* > 1), indicating that interpretations showing more of the activity were more likely to result in a correct diagnosis. A correct diagnosis was less likely to result from interpretations with higher magnification percentages (*adjusted* *OR* < 1).

### 6.3. Discussion

One of the major causes of medical morbidity and death is an incorrect cancer diagnosis (Zhang et al., 2019). Given the serious effects that diagnostic mistakes have on patients, it is critical to comprehend the underlying causes of these mistakes. It may be useful for both clinical and instructional purposes to investigate how pathologists interpret the digital slides and conduct their searches, and how these viewing behaviors affect the accuracy of their diagnoses. The relationship between the amount of time spent viewing the consensus ROI and diagnostic accuracy emphasizes how crucial it is to identify key regions and obtain high-power views of the histopathological features in these regions. We think this outcome can be utilized in future research and development as digital WSI and computer-aided diagnostic (CAD) tools continue to permeate training and clinical practice. A few computer models have been created based on how pathologists view breast histopathology photos when diagnosing patients (Mercan et al., 2014). Future adaptive tutoring systems can also keep track of student viewing behavior and adaptively direct new pathologists toward these aspects, assisting them in learning which image parts are crucial for making an accurate diagnosis.

## 7. Conclusion

This paper has described multiple different efforts to analyze skin biopsy whole slide images, including melanocyte and mitotic figure detection at the low level, semantic segmentation at the mid level, and diagnosis and viewing behavior analysis at the high level. Our deep learning results show that it is possible for computer programs to provide information that can aid dermatopathologists in their work. In a recent work (Nofallah et al., 2022a), we studied the impact of adding each tissue mask to the WSI in the classification of our dataset into diagnostic categories. Our experiments showed that including certain segmentation masks such as epidermal nests and dermal nests, specifically melanoma dermal nests, along with WSIs resulted in a better diagnosis performance. Our analyses of pathologists' viewing behavior identified the variables that were most important in the work of human pathologists as they diagnosed digital slides. The time is ripe for a fully-automated diagnosis aid that can support and advise pathologists as they perform their diagnoses.

## Data availability statement

The data analyzed in this study is subject to the following licenses/restrictions: dataset is restricted due to IRB and privacy concerns. Requests to access these datasets should be directed to JE, JElmore@mednet.ucla.edu.

## Author contributions

SN performed the work on mitosis finding and on semantic segmentation. KL did the work on melanocyte detection and WW on diagnosis. FG performed the study of pathologists' viewing behavior. JE and LS supervised the work. LS wrote the paper. All authors contributed to the article and approved the submitted version.

## Funding

The research reported in this study was supported by Grants R01CA151306, R01CA200690, and U01CA231782 from the National Cancer Institute of the National Institutes of Health, 622600 from the Melanoma Research Alliance, and W81XWH-20-1-0797 and W81XWH-20-1-0798 from the US Department of Defense. The funders had no role in the design and conduct of the study; collection; management; analysis; and interpretation of the data, preparation, review, or approval of the manuscript, nor the decision to submit the manuscript for publication.

## Conflict of interest

The authors declare that the research was conducted in the absence of any commercial or financial relationships that could be construed as a potential conflict of interest.

## Publisher's note

All claims expressed in this article are solely those of the authors and do not necessarily represent those of their affiliated organizations, or those of the publisher, the editors and the reviewers. Any product that may be evaluated in this article, or claim that may be made by its manufacturer, is not guaranteed or endorsed by the publisher.
